# Determination of a CrossFit^®^ Benchmark Performance Profile

**DOI:** 10.3390/sports9060080

**Published:** 2021-06-02

**Authors:** Nicole Meier, Stefan Rabel, Annette Schmidt

**Affiliations:** Institute for Sports Science, Faculty of Human Sciences, University of the Federal Armed Forces Munich, 85579 Neubiberg, Germany; nicolemeier2708@gmx.de (N.M.); Stefan.Rabel@unibw.de (S.R.)

**Keywords:** benchmark performance profile, CrossFit^®^ sport performance, high-intensity interval training, back squat performance

## Abstract

In the trend sport CrossFit^®^, international competition is held at the CrossFit^®^ Games, known worldwide as the definitive fitness test. Since American athletes are the best in the world regarding CrossFit^®^, there might be influencing factors on international competition performance. Here, we characterize the benchmark performance profile of American and German CrossFit^®^ athletes (n = 162). To collect the common benchmark performance by questionnaire, 66 male and 96 female CrossFit^®^ athletes (32.6 ± 8.2 years) participated in our survey in both nations. By comparing the individual performance variables, only a significant difference in total power lift performance by males was identified between the nations (*p* = 0.034). No other significant differences were found in the Olympic lift, running, or the “Girl” Workout of the Day (Fran, Grace, Helen) performance. Very large to extremely large (r = 0.79–0.99, *p* < 0.01) positive correlations were found between the power lift and Olympic lift variables. Further linear regression analysis predicted the influence of back squat performance on performance in the Olympic lifts, snatch (R^2^ = 0.76) and clean and jerk (R^2^ = 0.84). Our results suggested a dominant role of back squat performance in the assessment of physical fitness of CrossFit^®^ athletes.

## 1. Introduction

In the international competition of the trend sport CrossFit^®^, the CrossFit Games^®^, the athletes reach top performances every year [1]. Few previous studies have examined physiological variables that predict the performance at the CrossFit^®^ Games [2]. Despite Martínez-Gómez et al. associating athletes’ performances at the CrossFit^®^ Games Open 2019 with various power, strength, and aerobic markers [3], so far there are still no specific criteria that allow a prediction of the performance.

The training modality of CrossFit^®^, as varied, high-intensity interval training (HIIT), includes exercises from the main elements of gymnastics, weightlifting exercises, and cardiovascular activities, and is usually performed as the “Workout of the Day” (WOD), with the focus on constantly varying functional movements [4]. The CrossFit^®^ training concept aims to prepare athletes to perform a variety of workouts. Considering that the constant variation of workouts is an essential element of CrossFit^®^, in international competitions the WOD requirements are only announced to the athletes a few minutes before the competition [5]. The last-minute announcement of the WOD is an essential difference from other sports, as otherwise it is always known exactly which discipline will be performed in the next competition. Top performance in competition, as in any other sport, is only achievable after years of scheduled training, and requires continuous progression that is monitored in some manner during training [6].

Determining benchmarks and ascertaining performance variables of specific exercises and WODs can be applied for the progression monitoring [7]. Due to the constant variability of training, determination of benchmark performance is necessary, especially in CrossFit^®^. Since 2008, CrossFit^®^ athletes can use the online software “Beyond the Whiteboard” (BTWB) to collect benchmarks performance data and compare them with others.

For this purpose, particular benchmark workouts have been developed in CrossFit^®^, like “Hero” WODs or “Girl” WODs. These benchmark workouts must be performed to the same specifications every time [8]. For the “Fran” WOD, there are three rounds, including 21, 15, and 9 repetitions, for time, of 95/65-pound barbell thrusters (male/female) and pull-ups. The “Grace” WOD includes 30 repetitions of 135/95-pound clean and jerk (male/female) for time, and the “Helen” WOD includes 3 rounds of a 400 m sprint, 21 repetitions of 53/35-pound American kettlebell swing, followed by 12 pull-ups. In parallel, CrossFit^®^ also applies the performance variables in the most common weightlifting exercises for performance benchmarking. So, the one-repetition maximum (1-RM) of the power lifts (deadlift, back squat, bench press, and shoulder press) and the Olympic lifts (snatch and clean and jerk) are of special interest [8]. Previous studies investigated the predictive power for top rankings in the CrossFit Games^®^ 2013 and 2016 of the individual benchmark performance, and found no significant results [9,10]. The CrossFit Open^®^ is the main opportunity to qualify for the CrossFit Games^®^. Mangine et al. analyzed the primary success predictor at the 2018 CrossFit Open^®^, and concluded that body fat percentage had the most significant effect [2]. To predict the 19.1 CrossFit Open^®^ Workout and the WOD “Fran” performances, a further study concluded that absolute VO_2_ peak and CrossFit^®^ Total (one-repetition maximum tests for the squat, deadlift, and overhead press) might be influencing factors [11]. Moreover, it was observed that no German athlete has ever won the CrossFit Games^®^ since they began in 2007. On the other hand, the American participants are the best in the world regarding CrossFit^®^ [12]. However, no study has yet investigated significant differences in the athletes’ performance profile between both nations, so for the first time, we analyzed the variation between German and American CrossFit^®^ performances.

To find valid predictors of CrossFit^®^ performance, only a few studies have been conducted, and they showed conflicting results [13,14,15,16]. On the one hand, previous studies investigated the influence of the physiological variables of aerobic capacity and anaerobic power, and showed a significant influence on CrossFit^®^ performance [13,15]. On the other hand, studies have only demonstrated an effect of strength on the performance of the “Grace” and “Fran” WODs, but not for “Cindy” [14]. The examination of the CrossFit^®^ “Murph” challenge (1-mile run, 100 pullups, 200 pushups, 300 air squats, 1-mile run) showed that only the physiological parameter of body-fat percentage was significantly related to total “Murph” time [17]. Based on the results of Dexheimer et al. and Martinez et al., the back squat performance may be considered as a major predictor, so in one study, the back squat strength explained 42% of the variance of the “Fran” performance [15]. Martinez et al. found moderate to strong positive correlations between squat variables and performance in the different WODs [16]. In summary, not a single benchmark performance was found with high predictive power for the main CrossFit^®^ WOD performances. We hypothesize that considering the entire benchmark performance profile, rather than individual variables, will allow us to predict an athlete’s performance ability or compare the performance internationally.

Thus, the aim of our study is to analyze the benchmark performance profile of American and German CrossFit^®^ athletes in detail, and to investigate any significant differences. In addition, we wanted to verify individual parameters of the benchmark performance profile with our data that predicted specific CrossFit^®^ performance in previous studies [15,16].

## 2. Materials and Methods

Here, were report the characterization of international CrossFit^®^ athletes’ benchmark performance profile based on the benchmark data of American and German participants collected by using a questionnaire. We compared our results using the online benchmarking tool “BTWB” with over 60,000 data points of certain benchmark performances to determine the benchmark performance profile. Based on our sample, we asked whether significant differences occurred between nations and identified benchmark variables predicting others. Our results will allow CrossFit^®^ athletes to rank their performance internationally, identify deficiencies, and predict specific benchmark variables.

### 2.1. Participants

To characterize the benchmark performance profile of American and German CrossFit^®^ athletes, in this study, 162 CrossFit^®^ athletes (male = 66; female = 96) participated from the United States of America (n = 82) and Germany (n = 80). The average age of participants was 32.6 ± 8.2 years. On average, the athletes had a CrossFit^®^ experience of 3.4 ± 1.9 years, with a training scope per week of 6.6 ± 3.5 h (see Table 1). The study was conducted according to the guidelines of the Declaration of Helsinki and approved by the Institutional Ethics Committee of University of the Federal Armed Forces Munich, Germany.

### 2.2. Measures

The questionnaire contained 19 items for six overall metrics. Items 1–7 referred to anthropometric data, including gender, age, height, bodyweight, workout volume per week, workout frequency, and years of practice in CrossFit^®^. Item 8 required a focus on competition. The next items contained the current 1-RM for the common power lifts (bench press, deadlift, back squat, shoulder press), the 1-RM for the Olympic lifts (snatch and clean and jerk), and the running times for 400 m sprint or 1-mile. Finally, participants completed items 17–19 regarding their current times for the three most common “Girl” workouts, “Fran”, “Grace”, and “Helen”.

### 2.3. Procedure

The questionnaire was prepared in German and English, and both were validated for clarity for four weeks each. After validation, the English questionnaire was distributed in five CrossFit^®^ boxes around Austin (Texas, United States of America) to collect the American athletes’ data. In the same way, the German questionnaires were distributed in six CrossFit^®^ boxes around Munich and Ratisbon (Bavaria, Germany) to collect the data of the German athletes. To include more participants, the questionnaire was also placed online via the platform www.soscisurvey.de (accessed period from 15 October 2018 to 5 November 2018) and shared in social media groups of the participating CrossFit^®^ boxes. The survey period was four weeks for each. To further interpret the results, the sample’s performance profiles were compared using the “BTWB” benchmarking online tool, which includes a data set of millions of CrossFit^®^ athletes worldwide.

### 2.4. Statistical Analysis

Descriptive statistics were performed on participant characteristics (Table 1) and on performance data. All data are presented as mean ± standard deviation (SD). Potential outliers were inspected using a box plot and excluded for the description of the performance profiles. To obtain more informative benchmarks and arithmetic means, we also calculated percentile values for all performance variables from the sample and the online “BTWB” tool. Percentage thresholds of 1%, 10%, 25%, 50%, and 80% were determined to represent the different performance profiles by gender. Preliminary analyses were conducted to ensure there were no violations of the assumptions of normality and homogeneity of the variance. The normality was tested using the Shapiro–Wilk test and Q–Q plots, and the homogeneity of the variance using the Levene test. An independent sample t-test was conducted to compare the benchmark performance for American and German athletes. The Mann–Whitney U-test was performed when the assumption of normality or the homogeneity of the variance was violated. Simple Pearson’s r correlations were used to determine the associations between all benchmark performance data. R-values of 0.1, 0.3, 0.5, 0.7, and 0.9 were considered small, moderate, large, very large, and extremely large, respectively [18]. For each of the dependent Olympic-lift performance variables, a multiple regression model was created to analyze the influence of the independent power-lift performance variables. Each power-lift performance variable with significant influence (*p* < 0.001) was examined in a single linear regression model to create a predictive model of performance and to evaluate the R^2^ to determine the portion of explained variation. The regression assumptions were met by performing tests for multicollinearity using variance inflation factor values, homoscedasticity using a scatterplot of standardized residuals and predicted values, multivariate normality using Q–Q plots, and linearity using scatterplots. All analyses were conducted with the software package SPSS 25.0 (IBM, Armonk, NY, USA), and the level of statistical significance (α) was set at 0.05.

## 3. Results

The anthropometric data of the participants showed that the training scope per week (h) for males was 0.5 h higher than for females and 0.4 h higher for Germans in the national comparison. The CrossFit^®^ experience (years) average was 3.4 ± 1.9, without any major differences between the subgroups.

In Table 2, all performance data are shown by gender and nationality. When comparing the genders, we found that males’ total powerlift performance was 61% higher than that of females, and the total Olympic lift performance was 53% higher. Males reported faster times for all “Girl” WODs, despite the scaled weights. This effect was also evident for all run values, as shown in Table 2. The American athletes showed higher average values for all power-lift and Olympic-lift performances, without higher maximum ranges.

**Figure 1:**
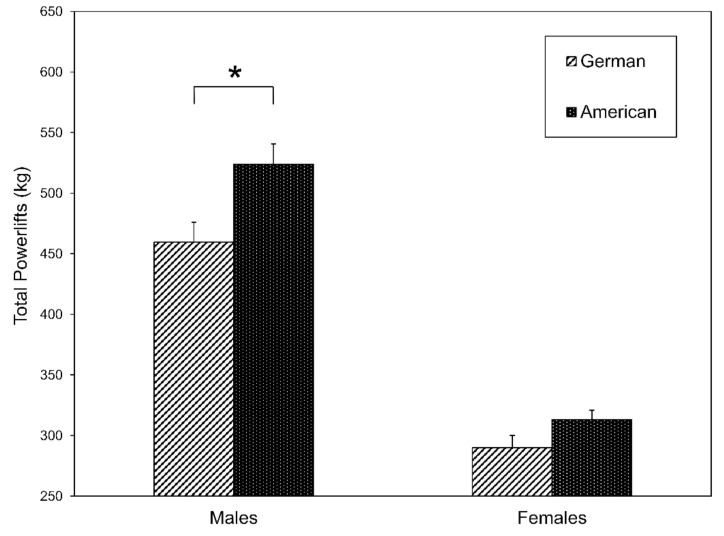
A significance difference was found between the total power-lift performances of American and German males (*p* = 0.034), but no significant difference was found for females. * *p* ≤ 0.05 for American and German Athletes.

We next studied whether there were significant differences in the performance benchmarks between the nations. The t-test for independent samples showed only a significant difference (54.5 kg) for the total power lift performance of Americans (523.9 ± 82.0 kg) and Germans (469.5 ± 105.8 kg) in males (t (64) = −2.17; *p* = 0.034), and no significant difference for females (t (94) = −2.33; *p* = 0.062)—see Figure 1. No other significant difference was observed in the Olympic lift performance and in the “Girl” WODs or running times between the nations.

**Figure 2:**
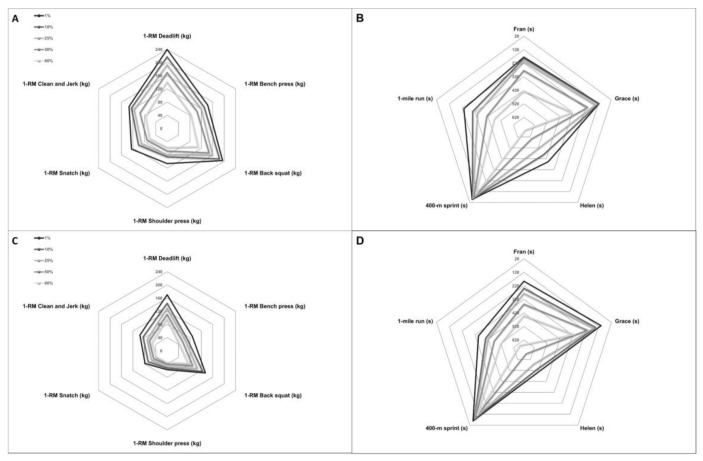
Benchmark performance profiles by gender. The lifting performance of males (**A**) and females (**C**) in comparison shows less total weight for females. The run and “Girl” Workout of the Day performance of males (**B**) and females (**D**) differed only partially.

The percentage of performance thresholds was calculated (Table 3) and graphically visualized in Figure 1 separated by gender to analyze the benchmark performance profile. According to percentage threshold values, the classification of the performance enabled a more precise description of the CrossFit^®^ athletes’ reachable physical fitness. So, females could move less weight in all weightlifting exercises in all performance groups. However, the proportion of the single weightlifting exercises was equally weighted between the genders. So, deadlift performance was the dominant exercise, with a bodyweight ratio of 2.0 for males and 1.7 for females, followed by the back squat performance, with a bodyweight ratio of 1.7 and 1.4, respectively. The bench press performance was not entirely as pronounced in females as in males, with a bodyweight ratio of 0.8 compared to 1.3 (for comparison, see Figure 2A,C). In descending order of expression, the subsequent weightlifting exercises and their bodyweight ratios for males and females were: clean and jerk (1.1 and 0.9), snatch (0.9 and 0.7), and shoulder press (0.8 and 0.6).

However, for the “Girl” WOD “Grace,” females achieved comparable top performances to males. The difference in mean times was only 1%. Nevertheless, the performance differences in the “Fran” WOD and the 1-mile time were less pronounced than in the “Helen” WOD and the 400 m run time. While females completed the “Fran” WOD an average of 70 s slower, the “Helen” WOD difference was an average of 84 s slower. Similar trends could be observed for the running performance, so the males ran the 400 m on average 25% faster, but the 1-mile only 18% faster.

To analyze the relationship between the benchmark performances, Pearson’s correlations were calculated (see Table 4). These significant correlations indicated that the power-lift performance was strongly related to the Olympic-lifting performance (r = 0.79–0.99; *p* < 0.01). Based on the data of this study, moderate to strong negative correlations between the weightlifting and the “Girl” WOD also were determined, but were partially nonsignificant (see Table 4). The performance in the “Helen” WOD was strongly related to the performance in the 400 m and 1-mile runs (r = 0.59 + 0.58; *p* < 0.01).

**Figure 3:**
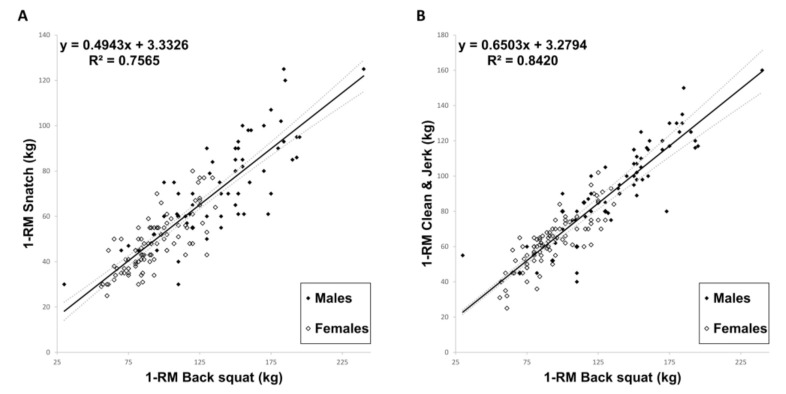
Relationship between the 1-RM back squat performance (kg) and the 1 RM snatch (kg) (**A**), and the 1-RM Clean and Jerk (kg) (**B**) by gender. The continuous line represents the line of best fit, and the dashed lines the 95% confidence intervals for each correlation.

Based on the Pearson’s correlation findings, multiple regression was calculated to predict the Olympic lift performance values, snatch, and clean and jerk, based on the single power-lift performance values. From the deadlift, bench press, back squat, and shoulder press performance values, only the back squat performance was a significant predictor of snatch and clean and jerk performance (*p* < 0.001). A simple linear regression was performed to predict participant’s snatch performance based on their back squat performance (see Figure 3A). A significant regression equation was found (F (1,160) = 497.081, *p* < 0.001), with an R^2^ of 0.756. Participants’ predicted snatch performance was equal to 3.333 + 0.494 (back squat performance) kg when back squat performance was measured in kilograms. Participants’ average snatch performance increased by 0.494 kg for each kilogram of back squat performance. To predict the clean and jerk performance on the back squat performance, a simple linear regression was calculated in the same way (see Figure 3B). The regression equation was also significant (F (1,160) = 852.916; *p* < 0.001), with an R^2^ of 0.841. The predicted clean and jerk performance was equal to 3.279 + 0.650 (back squat performance) kg. For each kilogram of back squat performance, the clean and jerk performance increased 0.650 kg.

## 4. Discussion

In this study, we characterized in detail the benchmark performance profile of American and German CrossFit^®^ athletes and compared the obtained data with thousands of available online data. We found only one significant difference, in the total power-lift performance of males between both nations. Based on our data, the power-lift and Olympic-lift variables showed very large to extremely large correlations. The back squat performance predicted 76% of the variance for the snatch performance, and even 84% of the variance for the clean and jerk performance.

To our knowledge, no studies have previously examined the benchmark performance profile of CrossFit^®^ athletes in detail. For the first time, we were able to describe the overall performance ability of CrossFit^®^ athletes and to identify differences between two nations. Mangine et al. presented normative scores for five common benchmark workouts (i.e., “Fran”, “Grace”, “Helen”, “Filthy-50”, and “Fight-Gone-Bad”) in a previous study, and observed that, on average, males achieved better scores than females for all WODs, despite scaled weights by gender [19]. However, the classification of performance by percentage thresholds in this study showed that females may well be able to achieve similar values to males in WODs without bodyweight exercises. We were able to show females of the 1% performance group achieved similar values for the “Grace” WOD consisting only of clean and jerk exercises (135/95 pounds for males/females) with scaled weights contrasted with the “Fran” and “Helen” WODs. Both WODs included the bodyweight exercise of pull-ups. Through all performance groups, females could not achieve similar values as males, confirmed by the data analysis using the online “BTWB” tool.

Finding only one significant performance difference between the two nations was surprising. This result did not confirm our assumption that the two nations’ different levels of success in the CrossFit Games^®^ would result in differences in fitness abilities. So, there could be other factors, such as social capital [20] or commercial environment, to achieve and sustain top athlete success as in other sports; e.g., in tennis [21].

Determining which variables predicted the performance of one of the best-known WODs, “Fran”, was also the purpose of previous studies. Leitão et al. showed that maximal and endurance strength training of thrusters was strongly related to “Fran” performance [22]. We can confirm moderate to strong negative correlations between weightlifting exercises and the “Girl” WODs “Fran”, “Grace”, and “Helen”, also in a multinational experimental group with a larger sample size, as in previous studies.

Our linear regression model was consistent with previous studies demonstrating back squat strength, explaining 84% of the variance for 1-RM clean and jerk performance and 76% of the variance for the snatch performance [14,23]. Thus, to the best of our knowledge, our regression model best describes the variance of snatch and clean and jerk performance of all existing studies regarding CrossFit^®^. Of note was our large sample size (n = 162), which distinguished our regression model from the noted experimental studies [15,16]. Martinez examined the influence of squat performance and performances in different WODs and found moderate to strong (r = 0.47–0.69, *p* < 0.05) positive correlations, as our data also showed [16]. This underlined squat as a major determinant of performance in CrossFit^®^.

However, CrossFit^®^ WODs often consist of multimodal exercises that include not only strength- and power-based actions, but also aerobic exercises like rowing or running. Thus, CrossFit^®^ is a complex training modality that requires different physical abilities (including stamina, flexibility, and agility). So, the interaction of different performances might play a role in the overall assessment of CrossFit^®^ athletes’ fitness abilities. For this reason, the total benchmark performance profile should be considered and combined with the assessment of other physical tests, such as the squat test from Martinez et al. [16].

While the present investigation provided some information about the benchmark performance profile and the relationship between the performance values, it was not without limitation. Since the present study was only a questionnaire survey, it is unknown whether the results could be reproduced in a performance test. However, the performance profile can be validated by comparing it with the data from the online “BTWB” tool. Due to the large size of the online data set, possible incorrect data did not have a significant impact.

The training concept of CrossFit^®^ intends to optimally prepare the athletes for unknown and unknowable challenges, and how they face them in competition. Identifying predictors for best performance in unknown challenges remains the major task of future CrossFit^®^ science. Our results confirmed the major role of back squat performance, and showed no differences in physical ability between German and American athletes. Further research should also apply cluster analysis, as shown by Peña et al., to find relationships between the outcome of a simulated CrossFit^®^ competition, anthropometric measures, and performance variables [24].

## 5. Conclusions

To better understand CrossFit^®^ performance, it is necessary to determine a CrossFit^®^ benchmark performance profile, as we have presented in this study. In future studies, the consistency of the benchmark performance profile could be confirmed by experimental data collection. In summary, the profile allows our results to rank CrossFit^®^ performance internationally, identify deficiencies, and predict specific benchmark variables.

## Figures and Tables

**Table 1:** Participant characteristics.

	All	Males	Females	American	German
n	162	66	96	82	80
Age (years)	32.6 ± 8.2	33.9 ± 9.0	31.7 ± 7.5	33.9 ± 8.5	31.2 ± 7.7
Height (cm)	172.4 ± 10.1	179.7 ± 7.5	167.4 ± 8.5	169.2 ± 8.8	175.7 ± 10.3
Weight (kg)	75.3 ±12.9	84.9 ± 10.1	68.7 ± 10.3	73.4 ± 12.7	77.3 ± 13.0
Training scope per week (h)	6.6 ± 3.5	6.9 ± 3.9	6.4 ± 3.3	6.3 ± 3.0	6.9 ± 4.0
CrossFit^®^ experience (years)	3.4 ± 1.9	3.3 ± 1.9	3.5 ± 1.9	3.5 ± 2.1	3.1 ± 1.8

Note: The values are expressed as mean ± standard deviation (SD).

**Table 2:** Performance data by gender and nationality.

Males	All	Range	American	Range	German	Range
n	66		24		42	
1-RM DL (kg)	172.1 ± 37.4	70–261	184.2 ± 31.6	136–261	165.1 ± 38.9	70–260
1-RM BP (kg)	106.3 ± 21.9	53–160	111.4 ± 18.4	80–159	103.4 ± 23.4	53–160
1-RM BS (kg)	140.8 ± 35.6	30–240	152.3 ± 26.5	93–193	134.1 ± 38.6	30–240
1-RM SP (kg)	70.2 ± 15.7	40–130	76.0 ± 17.2	57–130	66.9 ± 13.9	40–105
Total power lifts (kg)	489.3 ± 100.7	250–765	523.9 ± 82.0	393–715	469.5 ± 105.8	250–765
1-RM SN (kg)	74.2 ± 20.8	30–125	79.8 ± 19.0	52–125	71.0 ± 21.3	30–125
1-RM CJ (kg)	95.8 ± 25.8	40–160	100.4 ± 21.3	52–135	93.2 ± 27.9	40–160
Total Olympic lifts (kg)	170.0 ± 45.3	70–285	180.2 ± 38.7	104–260	164.2 ± 48.1	70–285
FR (s	310.4 ± 134.3	142–720	283.1 ± 116.1	142–480	325.0 ± 143.2	177–720
GR (s)	233.3 ± 101.2	115–430	257.0 ± 112.0	117–430	214.1 ± 92.6	115–390
HE (s)	611.2 ± 127.1	393–902	589.9 ± 150.8	393–902	625.8 ± 110.8	509–900
400 m (s)	76.3 ± 19.6	49–150	78.3 ± 24.1	51–150	75.1 ± 16.8	49–106
1 mile (s)	402.1 ± 80.7	234–570	401.0 ± 82.3	251–540	402.7 ± 81.2	234–570
**Females**						
n	96		38		58	
1-RM DL (kg)	114.4 ± 22.5	62–170	116.6 ± 22.1	62–170	111.1 ± 23.0	70–170
1-RM BP (kg)	54.3 ± 13.0	27–90	56.1 ± 12.3	27–84	51–7 ± 13.9	27–90
1-RM BS (kg)	92.6 ± 20.2	56–136	96.8 ± 19.4	56–136	86.1 ± 19.9	57–130
1-RM SP (kg)	42.8 ± 10.8	25–90	43.9 ± 11.1	25–90	41.0 ± 10.4	25–80
Total Powerlifts (kg)	304.1 ± 60.2	180–460	313.4 ± 57.2	180–424	289.9 ± 62.6	192–460
1-RM SN (kg)	48.2 ± 12.1	25–80	50.1 ± 11.6	29–77	45.3 ± 12.6	25–80
1-RM CJ (kg)	62.8 ± 14.8	25–102	64.4 ± 14.7	25–102	60.4 ± 14.8	35–95
Total Olympic lifts (kg)	111.0 ± 26.2	55–179	114.5 ± 25.4	55–179	105.7 ± 26.8	60–175
FR (s)	361.8 ± 112.7	142–641	346.3 ±109.8	142–640	390.3 ± 115.3	238–641
GR (s)	250.6 ± 171.2	100–1200	254.3 ± 187.8	116–1200	267.7 ± 107.0	100–482
HE (s)	698.8 ± 186.1	510–1621	673.5 ± 101.3	510–888	754.5 ± 318.0	532–1621
400 m (s)	93.8 ± 20.9	45–188	94.1 ± 16.5	59–123	93.0 ± 30.4	45–188
1 mile (s)	474.1 ± 85.1	242–800	472.4 ± 64.8	358–720	479.2 ± 129.0	242–800

Note: the values are expressed as mean ± standard deviation (SD). Abbreviations: BP = bench press, BS = back squat, CJ = clean and jerk, DL = deadlift, FR = Fran, GR = Grace, HE = Helen, RM = repetition maximum, SN = snatch, SP = shoulder press.

**Table 3:** Percentage thresholds of benchmark performances by gender.

Males	1%	10%	25%	50%	80%
1-RM DL (kg)	240 (248)	218 (210)	193 (190)	170 (166)	143 (140)
1-RM BP (kg)	142 (161)	130 (134)	120 (120)	105 (102)	85 (84)
1-RM BS (kg)	194 (211)	184 (175)	160 (156)	148 (135)	110 (110)
1-RM SP (kg)	106 (102)	86 (84)	79 (75)	68 (66)	57 (57)
1-RM SN (kg)	125 (120)	100 (98)	90 (84)	70 (70)	60 (57)
1-RM CJ (kg)	134 (145)	125 (120)	115 (107)	95 (93)	75 (77)
FR (s)	175 (139)	184 (187)	204 (247)	274 (337)	424 (479)
GR (s)	115 (95)	119 (131)	142 (163)	203 (214)	322 (313)
HE (s)	393 (442)	455 (507)	515 (556)	602 (630)	682 (753)
400 m (s)	49 (54)	55 (62)	60 (68)	72 (76)	92 (90)
1 mile (s)	234 (312)	303 (351)	340 (378)	413 (416)	472 (482)
**Females**					
1-RM DL (kg)	170 (160)	145 (134)	130 (116)	111 (102)	98 (84)
1-RM BP (kg)	88 (84)	71 (66)	64 (59)	55 (50)	44 (41)
1-RM BS (kg)	134 (136)	125 (108)	107 (95)	90 (80)	75 (64)
1-RM SP (kg)	57 (57)	52 (48)	48 (43)	41 (38)	35 (32)
1-RM SN (kg)	77 (75)	66 (59)	55 (50)	47 (41)	37 (32)
1-RM CJ (kg)	95 (93)	84 (75)	70 (66)	62 (55)	52 (45)
FR (s)	186 (162)	238 (245)	276 (311)	355 (400)	439 (536)
GR (s)	100 (107)	143 (150)	155 (187)	206 (245)	309 (345)
HE (s)	510 (490)	532 (574)	578 (633)	672 (714)	750 (825)
400 m (s)	59 (65)	75 (77)	82 (84)	90 (95)	109 (116)
1 mile (s)	346 (361)	402 (408)	420 (445)	469 (497)	521 (584)

Note: reference percentage thresholds from the online tool “Beyond the Whiteboard” are in parentheses. Abbreviations: BP = bench press, BS = back squat, CJ = clean and jerk, DL = deadlift, FR = Fran, GR = Grace, HE = Helen, RM = repetition maximum, SN = snatch, SP = shoulder press.

**Table 4:** Pearson’s correlation among the performance variables.

	1-RM BP (kg)	1-RM BS (kg)	1-RM SP (kg)	Total PL (kg)	1-RM SN (kg)	1-RM CJ (kg)	Total OL (kg)	FR (s)	GR (s)	HE (s)	400-m (s)	1 mile (s)
1-RM DL (kg)	0.86 **	0.93 **	0.84 **	0.97 **	0.83 **	0.88 **	0.87 **	−0.47 **	−0.30 **	−0.39 **	−0.50 **	−0.48 **
1-RM BP (kg)	1	0.84 **	0.89 **	0.94 **	0.79 **	0.82 **	0.82 **	−0.44 **	−0.21	−0.31 *	−0.44 **	−0.38 **
1-RM BS (kg)		1	0.84 **	0.96 **	0.87 **	0.92 **	0.91 **	−0.54 **	−0.29 *	−0.40 **	−0.47 **	−0.43 **
1-RM SP (kg)			1	0.92 **	0.80 **	0.81 **	0.82 **	−0.43 **	−0.12	−0.37 **	−0.45 **	−0.41 **
Total PL (kg)				1	0.87 **	0.91 **	0.91 **	−0.50 **	−0.26 *	−0.40 **	−0.50 **	−0.46 **
1-RM SN (kg)					1	0.93 **	0.98 **	−0.54 **	−0.24 *	−0.39 **	−0.46 **	−0.41 **
1-RM CJ (kg)						1	0.99 **	−0.59 **	−0.31 **	−0.45 **	−0.51 **	−0.46 **
Total OL (kg)							1	−0.57 **	−0.28 *	−0.43 **	−0.50 **	−0.44 **
FR (s)								1	0.58 **	0.37 **	0.45 **	0.37 **
GR (s)									1	0.37 **	0.33 **	0.33 **
HE (s)										1	0.59 **	0.58 **
400-m (s)											1	0.81 **
1 mile (s)												1

Note: * significant correlation *p* < 0.05; ** significant correlation *p* < 0.01. Abbreviations: BP = bench press, BS = back squat, CJ = clean and jerk, DL = deadlift, FR = Fran, GR = Grace, HE = Helen, RM = repetition maximum, SN = snatch, SP = shoulder press.

## References

[1] CrossFit (2020). Finding the Fittest on Earth. CrossFit Games.

[2] Mangine G.T., Tankersley J.E., McDougle J.M., Velazquez N., Roberts M.D., Esmat T.A., VanDusseldorp T.A., Feito Y. (2020). Predictors of CrossFit Open Performance. Sports.

[3] Martínez-Gómez R., Valenzuela P.L., Alejo L.B., Gil-Cabrera J., Montalvo-Pérez A., Talavera E., Lucia A., Moral-González S., Barranco-Gil D. (2020). Physiological Predictors of Competition Performance in CrossFit Athletes. Int. J. Environ. Res. Public Health.

[4] Glassman G. (2004). What is CrossFit?. Crossfit J..

[5] Schlegel P. (2020). CrossFit^®^ Training Strategies from the Perspective of Concurrent Training: A Systematic Review. J. Sports Sci. Med..

[6] Petrik M. (2014). CrossFit Powerworkouts: Intensivtraining für Kraft & Ausdauer.

[7] Claudino J.G., Gabbett T.J., Bourgeois F., Souza H.D.S., Miranda R.C., Mezêncio B., Soncin R., Filho C.A.C., Bottaro M., Hernandez A.J. (2018). CrossFit Overview: Systematic Review and Meta-analysis. Sports Med. Open.

[8] Glassman G. (2003). Benchmark Workouts. CrossFit J..

[9] Barbieri J.F., Correia R.F., Castaño L.A.A., Brasil D.V.C., Ribeiro A.N. (2017). Comparative and correlational analysis of the performance from 2016 crossfit games high-level athletes. Man. Ther. Posturology Rehabil. J..

[10] Bellovary B., Drum S.N. (2014). A Performance Profile Related to Building Elite Fitness in Male Competitors. Med. Sci. Sports Exerc..

[11] Zeitz E.K., Cook L.F., Dexheimer J.D., Lemez S., Leyva W.D., Terbio I.Y., Tran J.R., Jo E. (2020). The Relationship between CrossFit^®^ Performance and Laboratory-Based Measurements of Fitness. Sports.

[12] CrossFit (2019). Welcome to the 2019 CrossFit Games Season. CrossFit Games.

[13] Bellar D., Hatchett A., Judge L., Breaux M., Marcus L. (2015). The relationship of aerobic capacity, anaerobic peak power and experience to performance in HIT exercise. Biol. Sport.

[14] Butcher S., Neyedly T., Horvey K., Benko C. (2015). Do physiological measures predict selected CrossFit^®^ benchmark performance?. Open Access J. Sports Med..

[15] Dexheimer J.D., Schroeder E.T., Sawyer B.J., Pettitt R.W., Aguinaldo A.L., Torrence W.A. (2019). Physiological Performance Measures as Indicators of CrossFit^®^ Performance. Sports.

[16] Martínez-Gómez R., Valenzuela P.L., Barranco-Gil D., Moral-González S., García-González A., Lucia A. (2019). Full-Squat as a Determinant of Performance in CrossFit. Int. J. Sports Med..

[17] Carreker J.D., Grosicki G.J. (2020). Physiological Predictors of Performance on the CrossFit “Murph” Challenge. Sports.

[18] Hopkins W.G., Marshall S.W., Batterham A.M., Hanin J. (2009). Progressive Statistics for Studies in Sports Medicine and Exercise Science. Med. Sci. Sports Exerc..

[19] Mangine G.T., Cebulla B., Feito Y. (2018). Normative Values for Self-Reported Benchmark Workout Scores in CrossFit^®^ Practitioners. Sports Med. Open.

[20] Whiteman-Sandland J., Hawkins J., Clayton D. (2018). The role of social capital and community belongingness for exercise adherence: An exploratory study of the CrossFit gym model. J. Health Psychol..

[21] Brouwers J., Sotiriadou P., De Bosscher V. (2015). Sport-specific policies and factors that influence international success: The case of tennis. Sport Manag. Rev..

[22] Leitão L., Dias M., Campos Y., Vieira J., Sant’Ana L., Telles L., Tavares C., Mazini M., Novaes J., Vianna J. (2021). Physical and Physiological Predictors of FRAN CrossFit^®^ WOD Athlete’s Performance. Int. J. Environ. Res. Public Health.

[23] Stone M.H., A Sands W., Pierce K.C., Carlock J., Cardinale M., Newton R.U. (2005). Relationship of maximum strength to weightlifting performance. Med. Sci. Sports Exerc..

[24] Peña J., Moreno-Doutres D., Peña I., Chulvi-Medrano I., Ortegón A., Aguilera-Castells J., Buscà B. (2021). Predicting the Unknown and the Unknowable. Are Anthropometric Measures and Fitness Profile Associated with the Outcome of a Simulated CrossFit^®^ Competition?. Int. J. Environ. Res. Public Health.

